# Why Do Durations in Musical Rhythms Conform to Small Integer Ratios?

**DOI:** 10.3389/fncom.2018.00086

**Published:** 2018-11-28

**Authors:** Andrea Ravignani, Bill Thompson, Massimo Lumaca, Manon Grube

**Affiliations:** ^1^Language and Cognition Department, Max Planck Institute for Psycholinguistics, Nijmegen, Netherlands; ^2^Artificial Intelligence Lab, Vrije Universiteit Brussel, Brussels, Belgium; ^3^Research Department, Sealcentre Pieterburen, Pieterburen, Netherlands; ^4^Department of Clinical Medicine, Center for Music in the Brain, Aarhus University, Aarhus, Denmark

**Keywords:** rhythm, music perception, scalar expectancy theory, neural oscillations, integer ratio

## Abstract

One curious aspect of human timing is the organization of rhythmic patterns in small integer ratios. Behavioral and neural research has shown that adjacent time intervals in rhythms tend to be perceived and reproduced as approximate fractions of small numbers (e.g., 3/2). Recent work on iterated learning and reproduction further supports this: given a randomly timed drum pattern to reproduce, participants subconsciously transform it toward small integer ratios. The mechanisms accounting for this “attractor” phenomenon are little understood, but might be explained by combining two theoretical frameworks from psychophysics. The scalar expectancy theory describes time interval perception and reproduction in terms of Weber's law: just detectable durational differences equal a constant fraction of the reference duration. The notion of categorical perception emphasizes the tendency to perceive time intervals in categories, i.e., “short” vs. “long.” In this piece, we put forward the hypothesis that the integer-ratio bias in rhythm perception and production might arise from the interaction of the scalar property of timing with the categorical perception of time intervals, and that neurally it can plausibly be related to oscillatory activity. We support our integrative approach with mathematical derivations to formalize assumptions and provide testable predictions. We present equations to calculate durational ratios by: (i) parameterizing the relationship between durational categories, (ii) assuming a scalar timing constant, and (iii) specifying one (of K) category of ratios. Our derivations provide the basis for future computational, behavioral, and neurophysiological work to test our model.

## Integer ratios and musical rhythm

What are *small integer ratios*, and what makes integer-ratio rhythms special? A *ratio* between two inter-onset-intervals (IOIs) is the division between two, usually adjacent durations. *Integer* ratios can be written as a fraction: 1.5 equals 15/10 or 3/2, but $\sqrt{2}$ for instance cannot be written as a fraction. An integer ratio is *small* if the result of the division can be written as a small integer number divided by another small integer number e.g., 2/3, but not 23/51 (Pikovsky et al., 2003; Strogatz, 2003).

**Figure 1:**
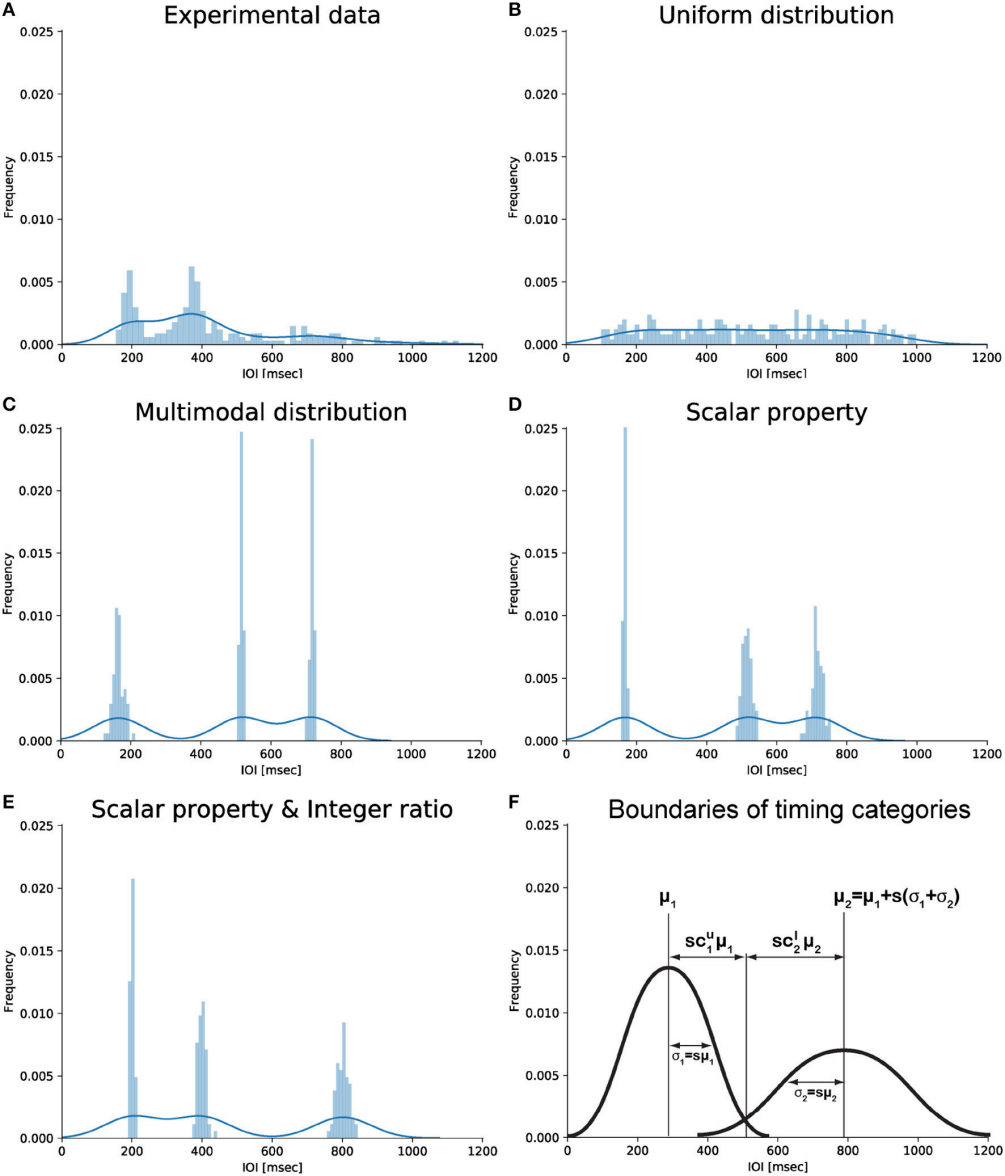
Graphical representation of different types of IOI distributions. **(A)** Empirical distribution of drumming data showing two peaks (slightly below 200 and 400 ms) consistent with the notion of integer ratio categories. Data from the last experimental generation of chain 2 in Ravignani et al. (2016). **(B)** Uniform distribution from 100 to 1,000 ms. **(C)** Multimodal distribution based on 3 randomly chosen centroids without further assumptions. **(D)** Multimodal distribution around the same 3 centroids assuming the scalar timing property. **(E)** Multimodal distribution assuming the scalar timing property and showing small integer ratios. Data in panels **(B–E)** are simulated; they were randomly sampled from several normal distributions, with total sample size as in **(A)**. **(F)** Schematic representation of potential parameters linking scalar timing and small integer ratios. Panel **(F)** was produced without simulated or experimental data. Notice how the x-coordinate of the intersection point between the two Gaussians can be parameterized as to $\mu_{1}+sc_{1}^{u}\mu_{1}$ (first Gaussian) and $\mu_{2}-sc_{2}^{l}\mu_{2}$ (second Gaussian). For more than two Gaussians, the intersection can be parameterized as $\mu_{k}+sc_{k}^{u}\mu_{k}$ (first Gaussian) and $\mu_{k+1}-sc_{k+1}^{l}\mu_{k+1}$ (second Gaussian). This parameterization is used in the derivations below.

A *rhythm*, by definition as used here, is a pattern of durations (London, 2004, p. 4) characterized by the succession of event onsets over time, in other words a series of IOIs. Auditory rhythms with small integer ratios between IOIs are common in the world's music (Essens and Povel, 1985; Toussaint, 2013; Savage et al., 2015). Psychological and neural research suggests that small integer-ratio rhythms allow a more accurate internal representation (Essens, 1986; Sakai et al., 1999), improved deviance detection (Jones and Yee, 1997; Large and Jones, 1999), enhanced memory (Deutsch, 1986; Palmer and Krumhansl, 1990) and reproduction (Povel and Essens, 1985; Essens, 1986), and better synchronization (Patel et al., 2005). The distortion of near-integer ratios toward integer ones (or their harmonics) reported in behavioral (Fraisse, 1982) and neurophysiological studies (Motz et al., 2013) further supports the idea of small ratios acting as “attractors” (Gupta and Chen, 2016). This idea has recently received support from studies of iterated learning and reproduction. When humans reproduce an initially randomly-timed rhythmic sequence, and this process is repeated in a cascade fashion within one or across several individuals, the sequence is subconsciously reshaped to be composed of IOIs related by small integer ratios (Figure 1A; c.f. Polak et al., 2016; Ravignani et al., 2016, 2018; Jacoby and McDermott, 2017).

Why do rhythms (i.e., patterns of durations) tend to exhibit small integer ratios? Why are humans drawn to rhythms with such a peculiar mathematical property, in both perception and production? Does this property reflect a special quirk of music perception and/or motor sequencing, or could it be explained by domain-general aspects of cognition? Can we explore these alternatives through mathematical formalism? Here, we explore mathematically the possibility that the human bias toward small integer ratios may be explained by a combination of scalar expectancy and categorical perception.

We begin by outlining the relevant classical frameworks for human timing, and go on to summarize the evidence in support of the small-integer ratio bias in rhythm perception. We then present our proposal linking the frameworks to the bias through mathematical formalisms. Specifically, we draw on the scalar property of time interval estimation to formulate a simple model of categorical perception that may result in an integer ratio bias (Figure 1), and link this to neural oscillations. We conclude by briefly discussing the merits and limitations of our model and outlining future goals.

## Psychophysical and oscillatory approaches

Two major theoretical approaches, among several, have been suggested to account for the mechanisms behind human timing (Wing and Kristofferson, 1973a,b; Getty, 1975; Meck, 1996; Church, 1999; Grondin, 2001, 2010; Mauk and Buonomano, 2004; Karmarkar and Buonomano, 2007; Ivry and Schlerf, 2008; Allman et al., 2014; Merker, 2014). The most influential and empirically tested psychoacoustic model is the “scalar expectancy theory” (Wearden, 1991; Allman and Meck, 2011). Psychophysical research shows that human timing often follows Weber's law (Bizo et al., 2006): the error for an interval duration being timed is proportional to the duration of that interval. One perception-based formulation states that the ratio between the just-noticeable difference (JND) and the duration of a reference stimulus is constant across stimulus length (Grondin, 2001). In another formulation, the coefficient of variation (standard deviation divided by mean) in estimating durations is constant across durations (Figure 1D; Gibbon, 1977).

Another relevant approach to timing mechanisms comes from neuroscience and physics. It suggests that neural oscillations entrain (or even “resonate”) with the periodicity of external stimuli at multiple time-scales (Buzsaki, 2006; Large, 2008; Arnal and Giraud, 2012; Gupta, 2014; Aubanel et al., 2016; Celma-Miralles et al., 2016). Specifically, it states that phase and frequency of neural oscillations entrain with the phase and frequency of external events at multiple metrical levels. For instance, processing a metronome beat will induce low-frequency oscillations and/or power fluctuations in high-frequency oscillations following the periodicity of the beat, plus its multiples or divisors. Critically, the stability of the connection between two or more active neural oscillations, i.e., the “resistance” to external perturbations, depends on the ratio of their periods (e.g., 1:1, 2:1, 2:3). Small integer ratios typically confer greater stability. This may explain the perceptual advantage for integer-ratio stimuli over more complex metrical patterns (Large and Kolen, 1995). Other frameworks state that specific neurons or neural channels are tuned to particular durational intervals or tempi (Merchant et al., 2013; Bartolo et al., 2014).

## Iterated drumming experiments: small integer ratios as cognitive attractors

Recent behavioral research investigated human priors for durations in rhythmic patterns (Ravignani et al., 2016, 2018; Jacoby and McDermott, 2017). Participants were given drumming sequences to reproduce to the best of their ability. The patterns produced were presented to the same or a new participant in an iterative procedure. Strikingly, “first-generation” participants were given completely random patterns, and “last-generation” participants produced rhythms exhibiting small integer ratios, in line with previous work on e.g., bimanual tapping (Peper et al., 1991, 1995a,b; Peper and Beek, 1998).

Specifically, participants were presented with sequences of IOIs sampled from a uniform distribution *U* (e.g., Figure 1B). As the patterns were transmitted through “chains of reproductions,” (Ravignani et al., 2016, 2018; Jacoby and McDermott, 2017), distribution *U* converged toward a distribution *D*: a human observer's posterior distribution of IOIs (e.g., Figure 1A). This distribution is multimodal, and the modes are related by small integer ratios, a universal property of human musical cultures (Ravignani et al., 2016; Jacoby and McDermott, 2017).

Here we aim to explain the distribution *D* via established psychophysical principles, none of which explicitly entail small-integer ratios. In other words, is the integer ratio bias a perceptual primitive in itself, or might it arise from the interaction of more fundamental primitives? Jacoby and McDermott (2017) related a theoretically hypothesized prior with built-in integer ratios to an empirically estimated prior, showing that these were aligned. Here, we investigate whether it is possible to derive a prior with similar properties by not building in the integer-ratio, but by combining empirically founded principles of timing with a minimum of assumptions (and room for refinement by future testing).

## Probabilistic inference for interval ratio categories

Our concrete question is: Under which conditions will a distribution *G* show small-integer ratios, without having built these ratios into our model?

Without any assumptions, distribution *G* would equal the uniform IOI distribution *U in expectation*. In other words which results on basic mechanisms of rhythm perception and production allow us to turn *U* into *G*? Below, we make four assumptions based on psychophysical evidence and reduce the number of free parameters in the model drastically with little loss of generality. We begin by elaborating on previous formalizations to make relevant assumptions explicit and comparable.

## Assumption 1: categorical timing

An *n*-event rhythm defines a sequence of IOIs ***d*** = (*d*_1_, …, *d*_*n*−1_) and of ratios ***r*** = (*r*_1_, …, *r*_*n*−2_), such that *r*_*i*_ = *d*_*i*+1_/*d*_*i*_. Perception of a rhythm ***r***induces a representation ***z*** = (*z*_1_, …, *z*_*n*−2_), with a strong tendency to categorize. The vector ***z*** is a sequence of a small number of unique phenomenal interval-ratio *categories* that represent the observed data ***r***. More specifically, the notation *z*_*i*_ = *k* identifies that interval ratio *r*_*i*_ is attributed to phenomenal category *k* (Ravignani et al., 2018). Whilst not used explicitly in our calculations, ***z*** formalizes the first key assumption: the processing of rhythmic sequences recruits a categorical interpretation of time intervals from a continuous stream of events (Clarke, 1987; Schulze, 1989; Desain and Honing, 2003). Behavioral evidence shows that also human motor timing is categorical: participants tapping produce IOI distributions with distinct peaks reflecting underlying durational *categories* (Collyer et al., 1994). This suggests that the distribution *G* can be approximated as a multimodal mixture of normal distributions (Figure 1C), rather than a uniform distribution (Figure 1B). A small number of durational categories naturally results in a small number of ratio categories. For the perception of a rhythmic sequence as a whole, we would argue that the perceived durations be transformed toward forming small ratios, as supported by iterated drumming experiments (Jacoby and McDermott, 2017), “ideally” into integer multiples of the smallest unit. Whilst categorical timing may appear to be a simplifying psychological concept (Schulze, 1989; Drake and Bertrand, 2001; Desain and Honing, 2003; ten Hoopen et al., 2006) based on behavioral observations, it may not be that far off neural reality. The notion of durational categories relate to basic durational tuning properties of premotor neurons recorded in non-human primates (Merchant et al., 2013). For instance, categories can be mapped to interval tuning in the premotor neurons of monkeys performing a synchronization continuation task (Merchant et al., 2013). Here, the distribution of preferred intervals could be viewed as a prior, although this distribution is multimodal, rather than bimodal as in Merchant et al. (2013). In addition, human neuroimaging work showed specific activation patterns for the perceptual processing of integer interval ratios (Sakai et al., 1999). Moreover, sequences of small integer ratios may induce a metrical beat by the hierarchical organization of periodicity at two or more levels, i.e., the occrurence of an accent at a multiple small integer of the shortest time unit at the next higher level (Povel and Essens, 1985). Metrical structure is thus a higher, multi-level demonstration of the psychological prior toward small-integer ratios, that affords accurate reproduction (Povel and Essens, 1985). Moreover, the perceptual timing of rhythms with such a metrical beat is more accurate, their subjective percept “catchier” and their recognition more robust against temporal scaling, i.e., speeding up or slowing down the tempo, as the pattern is processed as one coherent whole rather than a series of time intervals, in contrast to rhythms that feature small integer ratios but no metrical beat (Grube and Griffiths, 2009).

## Assumption 2: bayesian inference over gaussian categories

A general assumption in rhythm research is that perceptual timing can be described as a process combining prior beliefs with sensory input. One way to capture this mathematically is to model time perception as Bayesian inference (Jazayeri and Shadlen, 2010; Cicchini et al., 2012; Merchant et al., 2013; Pérez and Merchant, 2018). Whilst our analysis relies on the nature of the prior rather than how it is deployed during perceptual interpretation, taking a Bayesian viewpoint is useful. It lets us express a prior distribution as an inductive bias (Thompson et al., 2016) and has been successfully applied in previous models of time interval estimation (e.g., Jazayeri and Shadlen, 2010; Cicchini et al., 2012). Employing Bayesian inference, we can characterize participant behavior as attributing a categorical representation to interval ratio *r*_*i*_ according to the distribution *p*(*z*_*i*_ = *k*|*r*_*i*_) ∝ *p*(*r*_*i*_|*z*_*i*_ = *k*)*p*(*z*_*i*_ = *k*). Our focus is the prior distribution over categories, *p*(*z*_*i*_ = *k*), equivalently *G*. Alternatively, it would be possible to model learners' assumptions about a likelihood distribution as a source of bias (e.g., Jazayeri and Shadlen, 2010; Cicchini et al., 2012).

Jacoby and McDermott (2017) recently modeled *n*-interval rhythms as single points in the *n-1* dimensional simplex, and formulated a multivariate-mixture prior over this space, assuming Gaussian models to underlie each of the mixtures. Namely, they formulated a multivariate *p*(***z***) directly. Our approach to the prior is closely related. Like Jacoby and McDermott (2017), we express the prior as a mixture of Gaussian components. However, our formulation treats an *n*-interval rhythm as a set of *n-1* independent samples from a *univariate multimodal distribution*, rather than a single multivariate sample. The two approaches essentially represent minor variants of the model for covariance of interval ratio categories. The assumption that the distribution *p*(***z***) has a Gaussian form should be tested in future work, but is in line with existing work and a fair first approximation.

We write the prior as a *K*-dimensional Gaussian mixture of interval ratio categories, and the data likelihood as i.i.d. Gaussian underlying these categories, such that the marginal distribution of interval ratios has the form:

(1) $$\begin{array}{r} p\left(r\right)=G\left(r\right)=\prod_{i=1}^{n-1}\sum_{k=1}^{K}\varphi_{k}N\left(d_{i};\mu_{k},\sigma_{k}\right) \end{array}$$

Here, the prior assigns to each Gaussian *k* = *1, …, K* a weight in the mixture, *φ*_*k*_, which determines its relative prominence as a category; a category mean μ_*k*_, which specifies the expected interval ratio underlying this category; and a category variance σ_*k*_. The assumption we make is that weights are constant: $\varphi_{k}=K^{-1}$ (corresponding to an equal number of observations in the Gaussians in Figures 1C–E). Whilst we hope to examine this assumption empirically in the future, we proceed under the most neutral assumption: no interval-ratio category is privileged.

## Assumption 3: a small number of sub-second categories

Assuming that our indexing of categories under the prior is strictly ordered by the category means, such that $\mu_{j}<\mu_{k}\underset{\;}{\Leftrightarrow }j<k$, we can immediately express our second empirical constraint on distribution *G*: only few categories exist (Desain and Honing, 2003; Motz et al., 2013; Ravignani et al., 2016, 2018). *K* is naturally limited by our approach to only model components for *small* integer ratios, and these are limited in number. Furthermore, we bound the range of category means μ_*k*_ from 200 ms (London, 2004, p. 35) to 1,000 ms (Shaffer, 1983; Desain and Honing, 2003; Buhusi and Meck, 2005). This constraint limits *K* to the largest number of categories such that no category mean exceeds 1,000 ms:

(2) $$\begin{array}{r} K=argmax_{k}\;\mu_{k}\;s.t.\;\mu_{k}\leq 1000\text{ for }k=1,\ldots ,K. \end{array}$$

## Assumption 4: scalar timing

So far, our assumptions constrain neither category means μ_*k*_ nor standard deviations σ_*k*_. Our final, perhaps most central assumption is that timing exhibits *scalar properties* in the sub-second time range considered here (Gibbon, 1977; Matell and Meck, 2000). Scalar timing drastically reduces the number of free parameters describing distribution *G*, by expressing category variances as a function of category means. The standard deviation of each category σ_*k*_ equals the mean μ_*k*_ multiplied by a constant, dimensionless factor *s* (Figure 1E):

(3) $$\begin{array}{r} \sigma_{k}=s\;\mu_{k}. \end{array}$$

Previous empirical reports estimated *s* to approximate *0.025* (Friberg and Sundberg, 1995; Madison and Merker, 2004).

## Linking categorical perception and scalar timing: how close can we get to integer ratio intervals?

All four assumptions are empirically based and independent of each other. Now, *G* can be further characterized by the degree of overlap between Gaussians composing the mixture. To formalize this, we assume each category *k* to intersect with its adjacent neighbors *k*−*1* and *k*+*1* at a distance proportional to $c_{k}^{l}$ and $c_{k}^{u}$ away from its mean μ_*k*_ (Figure 1F), which is a constant proportion of the standard deviation σ_*k*_. $c_{k}^{l}$ and $c_{k}^{u}$ parameterize the overlap between categories: they express how many standard deviations away from its mean μ_*k*_ the cluster *k* intersects the cluster *k*+*1*, and how many standard deviations away from its mean μ_*k*+1_ the cluster *k*+*1* intersects the cluster *k* (Figure 1F shows an example for *k* = 1,2).

Combining this idea of a parameterized overlap with scalar properties, each cluster *k* extends from $\mu_{k}-sc_{k}^{l}\mu_{k}$ to $\mu_{k}+sc_{k}^{u}\mu_{k}$. Under these assumptions, the distance between the means of two adjacent distributions (Figure 1F) can be written as

(4) $$\begin{array}{r} \mu_{k+1}-\mu_{k}=sc_{k+1}^{l}\mu_{k+1}+sc_{k}^{u}\mu_{k}, \end{array}$$

and their ratio as

(5) $$\begin{array}{r} r_{k}=\mu_{k+1}/\mu_{k}\;. \end{array}$$

Substituting (5) into (4) provides

(6) $$\begin{array}{r} r_{k}\mu_{k}-\mu_{k}=sc_{k+1}^{l}r_{k}\mu_{k}+sc_{k}^{u}\mu_{k}, \end{array}$$

which can be simplified and rewritten as

(7) $$\begin{array}{r} r_{k}=\left(1+sc_{k}^{u}\;\right)/{\left(1-sc_{k+1}^{l}\right)}_{}. \end{array}$$

Equation (7) requires, to be well-defined, that its right side is positive, namely

(8) $$\begin{array}{r} \;0<c_{k+1}^{l}\;<\frac{1}{s}{{\;}_{}}_{\;}. \end{array}$$

Operationally, the category means following from the constraints on *G* can be calculated using the recursion equation:

(9) $$\begin{array}{r} \mu_{k+1}=r_{k}\mu_{k}. \end{array}$$

The constraints structure the space of component Gaussians in the prior such that, by specifying μ_1_, we can compute μ_*k*_ for all *k* ≤ *K* using Equation (9) (Figure 1E).

These quantitative tools enable the formulation of several questions. Given our *post-hoc* knowledge that the prior is characterized by categories centered at small integer ratios, do the constraints we laid out structure the prior such that integer-ratio clusters are predicted by setting μ_1_ to the smallest possible integer ratio?

An alternative approach might be to assume that one ratio is e.g., $\frac{1}{2}$, and ask whether our equations imply small integer ratios for the remaining cluster centers. More generally, do the constraints laid out impose an integer ratio structure on the prior without assuming an integer ratio for any of the clusters, simply by setting *c*_*k*_ in a certain way?

## How do $c_{k}^{u}\;\text{a}n\text{d}\;c_{k}^{l}$ relate to μ_*k*_ ?

The x-coordinates for the intersection point, expressed as $\mu_{k}-sc_{k}^{l}\mu_{k}$ and $\mu_{k}+sc_{k}^{u}\mu_{k}$, can be substituted in the respective Gaussian probability density functions, equated to impose the condition of intersection on the y-axis (Figure 1F):

(10) $$\begin{array}{l} 2*log\left(s^{}\mu_{k}^{}{\;}_{}\right)+\frac{{\left(\left(\mu_{k}+sc_{k}^{u}\mu_{k}\right)-\;\mu_{k}\right)}^{2}}{s^{2}\mu_{k}^{2}{\;}_{}}\\ \;=\;2*log\left(s^{}\mu_{k+1}^{}{\;}_{}\right)+\frac{{\left(\left(\mu_{k+1}-sc_{k+1}^{l}\mu_{k+1}\right)-\;\mu_{k+1}\right)}^{2}}{s^{2}\mu_{k+1}^{2}{\;}_{}} \end{array}$$

which simplifies as:

(11) $$\begin{array}{r} {\left(c_{k}^{u}\right)}^{2}-{\left(c_{k+1}^{l}\right)}^{2}\;{=}_{\;}2log\left(\mu_{k+1}/\mu_{k}\right). \end{array}$$

Equation (11) means that the difference of squares between *c*'s is proportional to the logarithm of the ratio of the two means.

To make an example with actual numbers, if one substitutes μ_*k*_ = μ_1_ = 100*ms* and μ_*k*+1_ = μ_2_ = 200*ms* in (11), the equation becomes ${\left(c_{k}^{u}\right)}^{2}-{\left(c_{k+1}^{l}\right)}^{2}\;{=}_{\;}2log\left(2\right)$. Hence $r_{1}=\frac{\mu_{k+1}}{\mu_{k}}=2$, $c_{1}^{u}\approx 2.5$ and $c_{2}^{l}\approx 2.2$ are two approximate solutions (among the infinite possible ones) of this particular example.

As the right side of Equation (11) is always strictly positive, $c_{k}^{u}$ can never equal $c_{k+1}^{l}$. While this does not constitute a mathematical contradiction with our formulation (still leaving an infinite number of mathematically possible *c*'s), it is admittedly difficult to interpret psychophysically.

## Suggested experiments: MODELING and psychophysics

**Figure 2:**
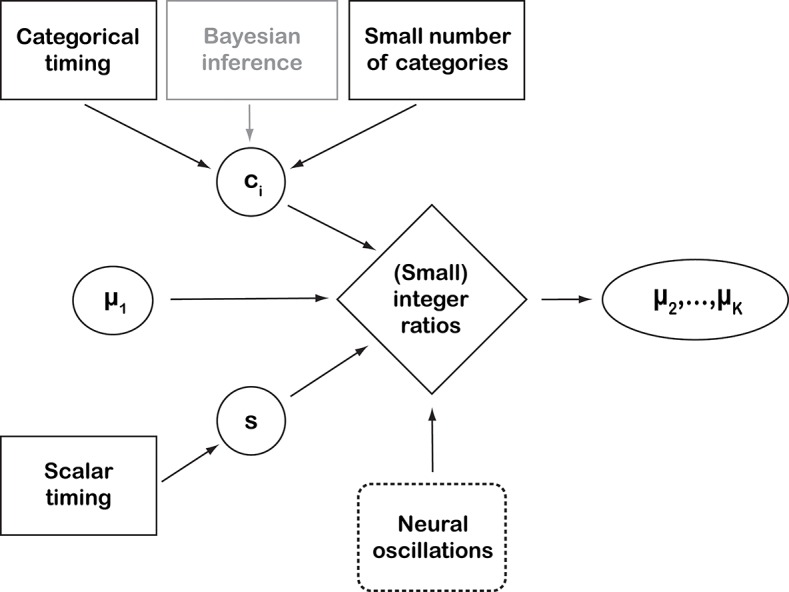
Schematic representation of the perspective introduced by this paper. Black solid-line boxes represent empirically supported assumptions. “Bayesian inference” is outlined in gray to indicate that it is used here as a working assumption and conceptual framework, rather than an empirically supported assumption on cognitive processes (Shi et al., 2013). “Neural oscillations” are dashed because they represent observed neural process whose connection with the other behavioral concepts has not been proven (yet). The quantitative parameters are: category means μ_*i*_, a scalar constant *s*, and *c*_*i*_, which is the abbreviation of $c_{i}^{l}$ and $c_{i}^{u}$, parameterizing the overlap between categories. The proposed way of representing rhythmic structure depends, among other factors, on the constancy of *r*_*k*_ (see main text). A deviation from this constancy would result in larger integer ratios, with the deviation accumulating over the categories when iterating equation (8). Empirical work (e.g., Ravignani et al., 2016; Jacoby and McDermott, 2017) has tried to operationalize the connection between the “mathematical perfection” of integer ratios and their empirical counterpart in a number of alternative ways. This perspective does not address how and when a real number is perceived as an integer ratio, leaving this as an empirical question for psychophysics research. In general, large integer ratios, and even irrational-number ratios, can be perceived as small integer ratios if close enough to one. For instance, 2^7/12^≈1.498307 is irrational (Coxeter, 1968) but close to 3/2. Virtually all pianos, today, employ this irrational number (1.498307) in their well-tempered tuning, which is “close enough” for human hearing to the integer ratio 3:2. At the same time, the “catchiness” of a rhythm also depends on small deviations from the integer ratios. For instance, delayed occurrences of expected beats even at varying levels of deviation from the underlying rhythms (together with the compensatory temporary speed-ups) are perceived as interesting, while a strictly regular rhythm will quickly appear dull.

Equations (7, 9) support a potential link between scalar timing and integer ratios, as they include the integer ratios *r*_*k*_ and the scalar constant *s* (Figure 2). These generative formulas can be implemented in computational simulations to explore the shape of the parameter space. Given specific values for parameters *s*, $c_{k}^{u}\;\text{and}c_{k}^{l}$, the equations will return a unique set of ratios: are these small integer ratios? Likewise, given one single integer ratio μ_1_, all other μ_*k*_ are determined by Equation (9): which values of μ_1_ result in ***r*** being integer ratios and *s*, $c_{k}^{u}\;\text{and}c_{k}^{l}$ being psychophysically plausible values?

The perspective we offer here creates the basis for expanding not only into theoretical but also empirical work on *s*, $c_{k}^{u}\;\text{and}c_{k}^{l}$. Experimental research can advance this approach by estimating *s*, $c_{k}^{u}\;\text{and}c_{k}^{l}$via Equation (7) or (11). Here, we treated the parameter *s* as an a priori known, one-valued constant (*s* = 0.025). To improve the model further, the variance of s might be estimated by replications of previous psychophysical experiments such as those by Friberg and Sundberg (1995) and Madison and Merker (2004). Values for $c_{k}^{u}\;\text{and}c_{k}^{l}$ can be estimated from experiments testing the perception (and misattribution) of durational categories.

## Limitations, discussion, and conclusions

We explore quantitative links between scalar timing and the human bias toward small integer ratios. The arguments we provide reduce the explanatory space to a few hypotheses. One possibility is that integer ratios are not a human cognitive primitive, but rather a simple by-product of other cognitive constraints, and their interaction.

Alternatively, the scalar timing framework might not be the most suitable one to explain the integer-ratio phenomenon of human rhythm. If one adopts oscillatory frameworks, integer ratios might simply arise from the oscillatory properties of brain activity, and so can scalar properties and categorical perception. Small integer ratios in particular would just reflect epiphenomena of harmonics of one oscillator or the interaction between two or more oscillators (Collyer et al., 1994; Strogatz, 2003; Buzsaki, 2006; Gupta, 2014; Merker, 2014; Gupta and Chen, 2016). Neural resonance to musical rhythm (Large, 2008), interval tuning (Merchant et al., 2013; Bartolo et al., 2014), and population clocks (Crowe et al., 2014; Gouvêa et al., 2015; Bakhurin et al., 2016; Merchant and Averbeck, 2017) present alternative timing mechanisms, documented by *in-vivo* recordings of neural populations and compatible with the observed small integer bias.

In any case, scalar timing and oscillatory theories are simplifications, i.e., approximate descriptions derived from confined experimental set-ups. Neurally and behaviorally, the dissociation or compatibility between scalar timing and oscillatory theories is more complex than it may appear in higher level cognitive theories, and only detailed neural models will enable us to define the actual underlying mechanisms.

## Author contributions

AR and BT conceived the idea and performed the mathematical derivations. All authors listed have made a substantial, direct and intellectual contribution to the work, and approved it for publication.

### Conflict of interest statement

The authors declare that the research was conducted in the absence of any commercial or financial relationships that could be construed as a potential conflict of interest.
